# Characterization of Neonicotinoid Metabolites by Cytochrome P450-Mediated Metabolism in Poultry

**DOI:** 10.3390/toxics12080618

**Published:** 2024-08-21

**Authors:** Adisorn Dam-on, Collins Nimako, Sittinee Kulprasertsri, Yoshinori Ikenaka, Yared B. Yohannes, Shouta M. M. Nakayama, Mayumi Ishizuka, Saranya Poapolathep, Amnart Poapolathep, Kraisiri Khidkhan

**Affiliations:** 1 Department of Pharmacology, Faculty of Veterinary Medicine, Kasetsart University, Bangkok 10900, Thailand; adisorn.da@ku.th (A.D.-o.); saranya.po@ku.th (S.P.); amnart.p@ku.th (A.P.); 2 Laboratory of Toxicology, Department of Environmental Veterinary Sciences, Faculty of Veterinary Medicine, Hokkaido University, Kita 18 Nishi 9, Kita-ku, Sapporo 060-0818, Japan; conimako@gmail.com (C.N.); y_ikenaka@vetmed.hokudai.ac.jp (Y.I.); ybyared@gmail.com (Y.B.Y.); shouta-nakayama@vetmed.hokudai.ac.jp (S.M.M.N.); ishizum@vetmed.hokudai.ac.jp (M.I.); 3 One Health Research Center, Hokkaido University, Sapporo 060-0818, Japan; 4 Department of Farm Resources and Production Medicine, Faculty of Veterinary Medicine, Kasetsart University, Kamphaeng Saen Campus, Nakhon Pathom 73140, Thailand; sittinee.k@ku.th; 5 Water Research Group, School of Environmental Sciences and Development, North-West University, P.O. Box X6001, Potchefstroom 2531, South Africa; 6 Translational Research Unit, Veterinary Teaching Hospital, Faculty of Veterinary Medicine, Hokkaido University, Sapporo 060-0818, Japan; 7 School of Veterinary Medicine, The University of Zambia, Great East Road, P.O. Box 32379, Lusaka 10101, Zambia

**Keywords:** neonicotinoids, metabolites, avian, metabolism, cytochrome P450

## Abstract

Neonicotinoids, a neuro-effective class of insecticides, are heavily applied in agricultural activities worldwide. Poultry can be exposed to neonicotinoids by several routes, but the knowledge of neonicotinoid’s metabolism in poultry and its associated interspecies differences is highly limited. Hence, this study aims to investigate the species differences in metabolite formations, as well as cytochrome P450 (CYP)-dependent metabolism of four major neonicotinoid compounds, acetamiprid, imidacloprid, clothianidin, and thiamethoxam, in poultry. In vitro biotransformation assays using hepatic microsomes of chicken, ducks, geese, quails, and rats were conducted. Metabolites of neonicotinoids were then screened by LC/Q-TOF and quantified by LC/MS/MS. The results revealed an existence of interspecies differences in the formations of *N*-[(6-chloro-3-pyridyl) methyl] -*N*-methyl acetamidine (IM-1-5) of acetamiprid and dm-clothianidin of clothianidin between chicken and other species. In addition, the greatest CYP activities in the metabolism of most neonicotinoid substrates, such as acetamiprid to dm-acetamiprid, imidacloprid to hydroxylated-imidacloprid and imidacloprid-olefin, clothianidin to dm-clothianidin, and thiamethoxam to clothianidin, were found in chicken. These results suggested that the CYPs in chicken may have a greater capacity for metabolism of neonicotinoids compared to other poultry. This study further revealed that the maximum intrinsic clearance of dn-imidacloprid and dn-clothianidin in ducks may be superintended by CYP-mediated nitro-reductions of imidacloprid and clothianidin. Further studies employing CYP recombinant enzymes may be required to elucidate the specific CYP isoforms that may be involved in neonicotinoid metabolism in avian species.

## 1. Introduction

Neonicotinoids, a class of neuroactive insecticides, are extensively used in monitoring pests in several agricultural crops [1]. Since the chemical structures of neonicotinoids are similar to nicotine, these compounds affect the central nervous system by binding to nicotinic acetylcholine receptors, leading to excitation and death of insects [2,3]. Because of their high water solubility and persistence in soil (half-lives ranging from 3 to >1000 days), neonicotinoids can be transported to many environmental compartments outside of their original application zones and may harm non-target creatures [4,5]. Acetamiprid, imidacloprid, clothianidin, and thiamethoxam are the most often detected neonicotinoids in various types of environmental matrices such as fruits, vegetables, soil, and water [1,6,7]. Thus, the presence of these neonicotinoids in the environment may present significant risks to both human and animal health.

Avian species are globally distributed in a variety of environments and climates. They can be exposed to chemicals, such as heavy metals, dioxins, and pesticides, by several routes [8,9]. In recent years, many studies have raised concerns about the potential adverse impacts of neonicotinoids on bird species [10,11,12]. Hallmann et al. [13] revealed that insectivorous bird populations tended to decline after the introduction of imidacloprid to the Netherlands. Similarly, in the United States, the increased usage of neonicotinoids between the years 2008 and 2014 caused a notable decline in avian biodiversity [10]. In addition, a previous study suggested that wild songbirds feeding on the imidacloprid-coated seeds had increased risks of mortality or exhibited significant loses in breeding chance [12].

The family of membrane-bound hemoproteins known as cytochrome P450s (CYPs) carries out various oxidative reactions on a diverse range of substrates [14]. Phase I metabolism of neonicotinoids is mainly based on these enzymes in the liver [15]. A previous in vitro analysis using human CYP recombinant enzymes indicated that CYP3A4 and CYP2C19 are key isoforms for the metabolism of thiamethoxam to clothianidin and clothianidin substrate to desmethyl-clothianidin [16]. In addition, Zhao et al. [5] suggested that the mechanism of toxicity of neonicotinoid pesticides may be involved in the impairment of CYPs. The study on the CYP metabolism of neonicotinoids using microsomes of dogs, cats, humans, and rats revealed that the difference in their kinetics and metabolite formation mainly depends on species and neonicotinoid substrates. For example, rats have a high oxidation rate of imidacloprid to 4OH-imidacloprid, while cats and humans showed the lowest formation of dm-clothianidin in the clothianidin metabolism [17]. In avian species, CYPs are among the most essential enzymes in the metabolism of pesticides, and the activity of CYPs varies by sex, age, and strain [6,18,19]. The total amounts and CYP activity in the avian livers are either slightly lower than or equivalent to those of mammals [18]. An in vitro and in silico investigation on avian CYP1-3 genes in chicken, zebra finch, and turkey discovered several notable characteristics of avian CYP1-3 genes and suggested that CYP2C45 may play a prominent role in the metabolism of xenobiotics in chicken [20]. Again, previous studies have indicated that several metabolites of neonicotinoids may be more toxic to animals than the parent compounds [21,22,23]. For instance, the thiacloprid-amide (metabolite of thiacloprid) and desnitro-imidacloprid (metabolite of imidacloprid) had higher cytotoxicity potency on fish leukocytes than their parent substances [24]. Nauen et al. [25] and Benzidane et al. [26] suggest that clothianidin (metabolite of thiamethoxam) has a high affinity to nicotinic acetylcholine receptors that could be as toxic as the parent compound. These emphasize the need for a more comprehensive understanding of interspecies differences in neonicotinoid metabolism, especially detoxifying enzymes such as CYPs.

The aim of the current study was to elucidate the interspecies differences in metabolite formations and CYP activities following the metabolism of major neonicotinoid compounds such as imidacloprid, acetamiprid, clothianidin, and thiamethoxam among poultry species (chicken, ducks, geese, and quails) and rats. To the best of our knowledge, there is no existing information clarifying CYP-dependent metabolism of neonicotinoids, and its associated metabolite formations in poultry. Our findings provide a first piece of evidence of neonicotinoid accumulation, excretion, sensitivities and the plausible toxicities in avian species.

## 2. Materials and Methods

### 2.1. Chemicals

Acetamiprid, imidacloprid, clothianidin, and thiamethoxam (purity: ≥99.5%) were purchased from LGC Dr. Ehrenstorfer (Middlesex, UK). Magnesium chloride (MgCl_2_) and glucose 6-phosphate (G6P) were obtained from Sigma-Aldrich (St. Louis, MO, USA). Glucose 6-phosphate dehydrogenase (G6PDH) and β-Nicotinamide adenine dinucleotide phosphate (β-NADPH) were purchased from Oriental Yeast Co., Ltd. (Tokyo, Japan) and Calbiochem (Darmstadt, Germany), respectively. Acetonitrile, methanol, ammonium formate, and formic acid were obtained from Kanto Chemical Co., Inc. (Tokyo, Japan). The BCA protein assay kit was purchased from Thermo Fisher Scientific (Rockford, IL, USA). The standards of target neonicotinoid metabolites (Table S1) were obtained from Wako Pure Chemical Co. (Osaka, Japan) or synthesized at Toho University (Chiba, Japan). Internal standards of acetamiprid (acetamiprid-D3), imidacloprid (imdacloprid-D4), clothianidin (clothianidin-D3), and thiamethoxam (thiamethoxam-D4) were purchased from Hayashi Pure Chemical Ind., Ltd. (Osaka, Japan). Internal standards of 4-hydroxy-imidacloprod (4OH-imidacloprod-13C-15N2), 5-hydroxy-imidacloprod (5OH-imidacloprod-13C-15N2), imidacloprid-olefin (imidacloprid-olefin-13C-15N2), 6-chloronicotinoic acid (6-CNA-13C6), *N*-desmethyl-acetamiprid (dm-acetamiprid-D3), and desmethyl-clothianidin (dm-clothianidin-13C-15N2) were acquired from Cambridge Isotope Laboratories, Inc. (Tewksbury, MA, USA).

### 2.2. Samples and Microsome Preparation

All experimental protocols and animal-handling procedures were performed following the guidelines for animal experiments and approved by the Animal Ethics Research Committee of the Faculty of Veterinary Medicine, Kasetsart University, Bangkok, Thailand (Approval number: ACKU66-VET-026). Liver samples of chickens (*Gallus domesticus*, *n* = 5), ducks (*Anas platyrhynchos*, *n* = 5), geese (*Anser domesticus*, *n* = 3), quails (*Coturnix coturnix*, *n* = 5), and rats (*Rattus norvegicus*, *n* = 3) were obtained from the previous study [27]. The information on studied animals used to obtain liver microsomes is provided in Table S2. The liver microsomal fractions used in this study were prepared by the method of Omura and Sato [28] with slight modifications. Briefly, liver sample was homogenized with 0.1 M potassium phosphate buffer (KPB, pH 7.4) and centrifuged at 9000× *g* at 4 °C for 20 min. The supernatant was collected and centrifuged twice at 105,000× *g* at 4 °C for 60 min. Microsomal pellets were homogenized with 0.1 M KPB and measured for protein concentrations using the BCA protein assay kit (Thermo Fisher Scientific, Rockford, IL, USA) before being frozen using liquid nitrogen, for storage at −80 °C until analysis.

### 2.3. In Vitro CYP Metabolism of Neonicotinoids

The assay of neonicotinoid metabolism using liver microsome was modified from that of a previous study [17]. Since we had insufficient liver samples to produce the microsomes of geese and quails, the substrates of imidacloprid and clothianidin were not applied for those microsome reactions. However, microsomes of chicken, ducks, and rats were used for all substrate reactions. A mixture of 0.1 M KPB, MgCl_2_ (3 mM), G6P (5 mM), neonicotinoid substrate (acetamiprid or imidacloprid or clothianidin or thiamethoxam, 0.125–80 μM), and hepatic microsome (1 mg/mL) of each species was pre-incubated for 5 min in a thermo-shaker. Then, the reaction was initiated by adding a mixture of G6PDH (2 IU/mL) and β-NADPH (0.5 mM), and continuously incubating for 30 min. Then, 0.1% formic acid in acetonitrile was added to stop the reaction. After that, the reaction sample was placed on ice for 15 min before centrifugation at 12,000 g for 10 min. The supernatant (100 μL) was collected and then spiked with neonicotinoid internal standard mixtures (50 μL), then evaporated using a centrifugal evaporator (CVE-200D with UT-2000, EYELA, Tokyo, Japan). The dried sample was re-dissolved with 20% methanol (*v*/*v*) and transferred into vials for chemical analysis.

### 2.4. Metabolite Screening by LC/Q-TOF and Quantification by LC/MS/MS 

A liquid chromatograph quadrupole time-of-flight (6546 LC/Q-TOF; Agilent Technologies, Santa Clara, CA, USA) operated with a 1.8 μm Eclipse Plus C18 RRHD analytical column (2.1 × 150 mm; Agilent Technologies, CA, USA) at a flow rate of 0.25 mL/min was used in the screening test to search for chromatographic peaks with the exact molecular mass of the possible neonicotinoid metabolites (Table S3). The mobile phases were 10 mM ammonium formate in water (A) and 10 mM ammonium formate in MeOH (B). The gradient elution of mobile phase B was set as follows: 2% (0–1 min), 100% (1–30 min), continued 100% (30–39 min), and a 4 min post-run at 2%. An injection volume was 5 μL and the column temperature was maintained at 45 °C. The expected formulas of potential metabolites of the four neonicotinoids were derived from previous publications on neonicotinoid metabolites in environment samples [29,30] and in animals [22,31].

A liquid chromatography mass spectrometer (Ultivo Triple Quadrupole LC/MS; Agilent Technologies, CA, USA) equipped with a 2.6 μm Biphenyl 100 A LC column (150 × 2.1 mm; Kinetex, Phenomenex Inc., Torrance, CA, USA) was applied to quantify the target metabolites of acetamiprid, imidacloprid, clothianidin, and thiamethoxam (Table S1). For all analyses, mobile phase A consisted of 0.1% formic acid + 10 mM ammonium acetate in distilled water and mobile phase B consisted of 0.1% formic acid + 10 mM ammonium acetate in 100% MeOH. The injection volume of 5 μL, the flow rate of 0.40 mL/min with gradient elution, and a column temperature of 45 °C were used for all experiments. The ion signals were acquired with multiple-reaction monitoring (MRM) in positive ionization mode.

In the current study, the liver microsome was spiked with deuterium-labelled internal standards (ISs) of the target neonicotinoids before the extraction and purification processes. About 13 neonicotinoid compounds were quantified by the internal standard method. The calibration curves were created using 10-point calibration standards ranging from 0.05 to 20 ng/mL. Calibration curves were plotted using standard peak area/IS peak area ratios, and average coefficients of determination (r^2^) for the calibration curves were ≥0.995. The accuracy of the current analytical method was validated and determined using matrix-spiked neonicotinoid standards at two concentrations, 1 ng/mL and 10 ng/mL. The thirteen target compounds were detected with recovery rates ranging from 87 to 101% at 1 ng/mL, and 86 to 116% at 10 ng/mL. The precision of the analytical technique was confirmed by inter-day and intra-day analysis; and the relative standard deviations were found to be less than 15% for all the target compounds. In the current study, all the assays were performed in duplicates and the negative controls (the reaction mixture without substrate) were prepared for each sample.

### 2.5. Data Analysis

A Michaelis–Menten equation was performed to calculate kinetic parameters including maximum velocity (V_max_), Michaelis–Menten constants (K_m_), and V_max_/K_m_ ratio in the GraphPad Prism version 8.4.3 software for Windows (San Diego, CA, USA). Statistical analyses were conducted using the JMP Pro 17 software (SAS Institute, Carry, NC, USA). One-way ANOVA with post-hoc Tukey–Kramer multiple comparison test was used to compare the V_max_/K_m_ ratio among species. All results were presented as the mean ± standard deviation (SD). A *p*-value < 0.05 was considered statistically significant in all analyses.

## 3. Results and Discussion

### 3.1. Identification of Metabolites and Differences in Kinetics of Neonicotinoids among Species

**Figure 1:**
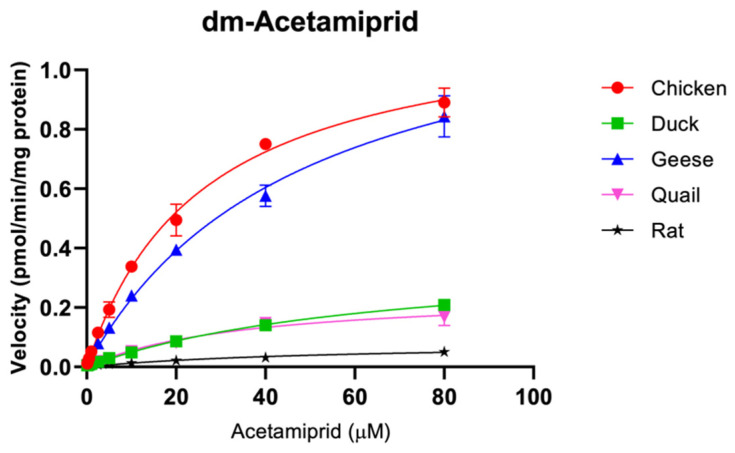
Michaelis–Menten plots for CYP activity (mean ± SD) of acetamiprid in chicken (red circle), duck (green square), goose (blue triangle), quail (pink triangle), and rat (black star) liver microsomes.

**Figure 2:**
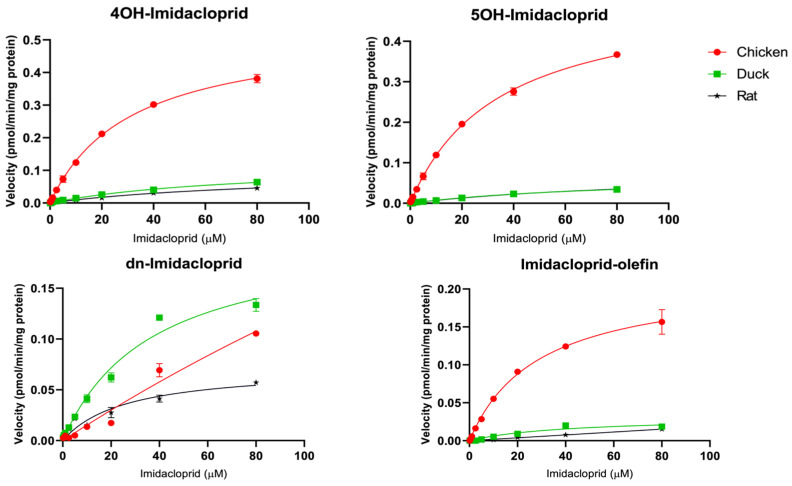
Michaelis–Menten plots for CYP activity (mean ± SD) of imidacloprid in chicken (red circle), duck (green square), and rat (black star).

**Figure 3:**
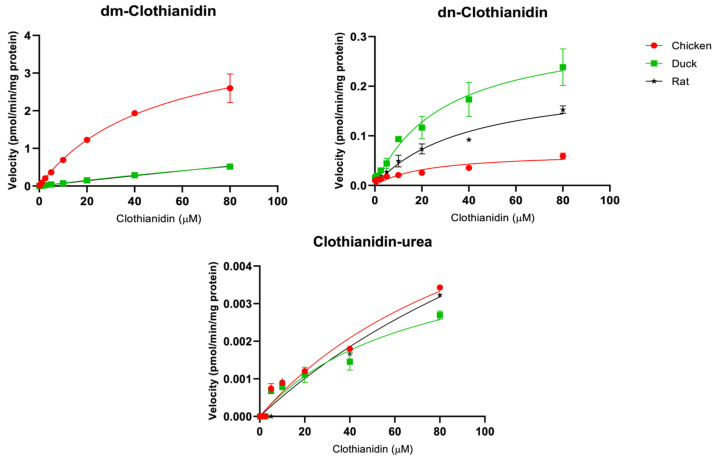
Michaelis–Menten plots for CYP activity (mean ± SD) of clothianidin in chicken (red circle), duck (green square), and rat (black star).

**Figure 4:**
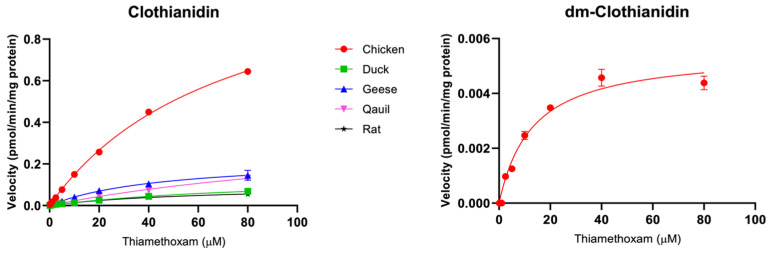
Michaelis–Menten plots for CYP activity (mean ± SD) of thiamethoxam in chicken (red circle), duck (green square), goose (blue triangle), quail (pink triangle), and rat (black star).

This study first scanned for the potential metabolites of predominantly used neonicotinoid insecticides, acetamiprid, imidacloprid, clothianidin, and thiamethoxam in various species using microsome mixtures (containing 800 µM of each of the target neonicotinoid substrates) and LC/Q-TOF techniques. Several neonicotinoid metabolites were formed in our current in vitro biotransformation assays (Table 1). Further investigations using LC/MS/MS techniques revealed significant differences in kinetic parameters such as the maximum rates of reaction (V_max_), enzyme-substrate affinities (K_m_), and intrinsic clearance (V_max_/K_m_), among the five avian species considered in the current study, chicken, ducks, geese, quails, and rats (Table 2 and Figure 1, Figure 2, Figure 3 and Figure 4).

#### 3.1.1. Acetamiprid

Metabolites of acetamiprid including *N*-desmethyl-acetamiprid (dm-acetamiprid or IM-2-1) and *N*-methyl(6-chloro-3-pyridyl) methylamine (IM-1-4) were found in all the studied species (Table 1). Among the two metabolites, IM-2-1 stood out as the major metabolite in chicken, ducks, geese, quails, and rats. These findings were similar to findings from previous exposure experiments in poultry and rats; however, the amount of IM-1-4 reported in the reference rat experiment was relatively small [32,33]. Whereas the *N*-[(6-chloro-3-pyridyl) methyl]-*N*-methyl acetamidine (IM-1-5) metabolite of acetamiprid was not detected in the reaction mixtures involving chicken microsome, this metabolite was clearly detected in microsomes of the other target species. Although IM-1-5 was generally transformed and detected in soil [34], our study revealed that this metabolite was also formed in ducks, geese, quails, and rats. Except for dm-acetamiprid, most known acetamiprid metabolites were hardly detected by LC/MS/MS in our current study. The Michaelis–Menten plots and kinetic parameters of dm-acetamiprid for all the target species are given in Figure 1 and Table 2. Significant differences in CYP activities in metabolism of acetamiprid to dm-acetamiprid were found among poultry species; the greatest clearance was found in chicken (46.5 ± 3.5 nL/min/mg protein), followed by geese (27.7 ± 0.5 nL/min/mg protein), quails (7.2 ± 0.2 nL/min/mg protein), ducks (5.7 ± 0.4 nL/min/mg protein), and rats (1.5 ± 0.1 nL/min/mg protein). Acetamiprid was rapidly metabolized by phase I enzymes, including CYP and aldehyde oxidase in the liver of mammals [35] and the acetamiprid demethylation to dm-acetamiprid was previously reported as the main acetamiprid metabolic pathway in rats [32,36]. Our study suggests that the specific CYP isoforms involved in the metabolism of acetamiprid found in chicken may differ from other target species.

#### 3.1.2. Imidacloprid

By comparing the CYP-mediated metabolism of imidacloprid among chicken, ducks, and rats, similar trends in imidacloprid metabolite formations were observed among the three species (Table 1). Liver microsomes of chicken, ducks, and rats could transform the imidacloprid substrate to hydroxylated imidacloprid (4OH- and/or 5OH-imidacloprid), desnitro-imidacloprid (dn-imidacloprid), and imidacloprid-olefin. For the hydroxylated imidacloprid, we could not separate 4OH-imidacloprid from 5OH-imidacloprid by LC/Q-TOF because both imidacloprid metabolites had similar molecular weights. Hence, we employed the LC/MS/MS technique to identify those metabolites, and our results indicated that 4OH-imidacloprid and 5OH-imidacloprid are the main metabolites for CYP-dependent metabolism of imidacloprid in chicken, ducks, and rats. In addition, imidacloprid-olefin and dn-imidacloprid were found as minor metabolites in chicken and ducks, but these metabolites were hardly detected in rats. Table 2 and Figure 2 show the differences in Michaelis–Menten plots and kinetic parameters of the four metabolites of imidacloprid among the three target species. Chicken had the maximum V_max_/K_m_ of 4OH-imidacloprid (17.1 ± 0.6 nL/min/mg protein), 5OH-imidacloprid (15.3 ± 0.0 nL/min/mg protein), and imidacloprid-olefin (7.5 ± 0.9 nL/min/mg protein). Similar clearance rates of 4OH-imidacloprid (1.6 ± 0.1 and 1.1 ± 0.3 nL/min/mg protein) and 5OH-imidacloprid (0.8 ± 0.1 nL/min/mg protein) were observed in ducks and rats. In the current study, the highest intrinsic clearance of dn-imidacloprid (5.5 ± 0.6 nL/min/mg protein) was found in ducks. Humans’ CYP3A4 and CYP2D6 are selective enzymes for formations of 5OH-imidacloprid and dn-imidacloprid [37,38]. The phylogenetic and synteny analyses suggested no clear ortholog of CYP3A genes between birds and humans [20]. In addition, Cai et al. [39] suggested that the gene structure of human CYP2D6 is similar to avian CYP2D49. In birds, although CYP activity is commonly comparable to or lower than that of mammals [18], the results of our study indicated that CYP3A and/or CYP2D49 in chicken and ducks may more efficiently metabolize imidacloprid to the 5OH-imidacloprid and dn-imidacloprid metabolites, compared to rats.

In mammals, the aldehyde oxidases are essential metabolism enzymes for nitro-reduction of imidacloprid to dn-imidacloprid [40,41]. Based on our results, the highest intrinsic clearance of dn-imidacloprid in ducks might have been instigated by the hepatic CYP enzymes that are capable of mediating the nitro-reduction pathway of imidacloprid. Since the dn-imidacloprid showed a higher affinity for nicotinic acetylcholine receptors than imidacloprid, this metabolite is predicted to be more toxic to mammals than the parent compound [41]. Hence, the greater formation of dn-imidacloprid in ducks suggests that ducks may be more susceptible to the toxicological impacts of imidacloprid, compared to the other studied species.

#### 3.1.3. Clothianidin

We found two metabolites of clothianidin including dm-clothianidin and clothianidin-urea after the in vitro metabolic assays of clothianidin with microsomes of chicken, ducks, and rats (Table 1). In addition to dm-clothianidin and clothianidin-urea, dn-clothianidin and desmethyl-desnitro-clothianidin (dm-dn-clothianidin) were detected by LC/MS/MS. Among these detected metabolites, dm-clothianidin was the most predominantly formed in avian species including chicken and ducks, while dn-clothianidin was the major metabolite in rats. The kinetic parameters and Michaelis–Menten plots for the CYP-dependent metabolism of clothianidin in chicken, ducks, and rats are shown in Table 2 and Figure 3. The intrinsic clearance of dm-clothianidin and dn-clothianidin was greatest in chicken (86.8 ± 12.6 nL/min/mg protein) and ducks (10.9 ± 0.2 nL/min/mg protein). Chicken, ducks, and rats showed no significant differences in the enzyme kinetics of clothianidin metabolism to clothianidin-urea. In humans, CYP2A6, CYP2C19, and CYP3A4 are suggested to be the key enzymes responsible for transforming clothianidin to dm-clothianidin [16]. In avian species, Watanabe et al. [20] suggested that xenobiotic-metabolizing CYPs, especially CYP2C and CYP3A, may be greatly involved in the evolution of avian-specific gene duplications. They further found out that CYP2C45 was the most greatly expressed CYP isoform in the liver of chicken and may play a central role in xenobiotic metabolism [20]. Our current findings strongly suggest that analogous CYP isoforms in avian species may have higher clothianidin demethylation ability than in rats.

#### 3.1.4. Thiamethoxam

The in vitro thiamethoxam metabolism assay using microsomes of chicken, ducks, and rats revealed the formation of various metabolites; these include clothianidin, clothianidin-urea, dm-clothianidin, *N*-desmethyl-thiamethoxam, and thiamethoxam-urea (Table 1). The major metabolite of thiamethoxam observed in the current target species was clothianidin. These findings are highly consistent with a previous in vivo exposure study of thiamethoxam [19], in which clothianidin and dm-thiamethoxam were identified as key metabolites of thiamethoxam in Japanese quails (*Coturnix japonica*). While the dm-clothianidin was earlier reported as a minor metabolite of thiamethoxam in quail species [19], the results of LC/Q-TOF and LC/MS/MS in our study indicated that this metabolite was formed only in chickens, but not in ducks, geese, and quails. Perhaps these contrasting results might have been instigated by differences in experimental species and thiamethoxam exposure doses. The Michaelis–Menten plots, V_max_, K_m_, and V_max_/K_m_ values of clothianidin and dm-clothianidin are presented in Figure 4 and Table 2. The fastest clearance of clothianidin was observed in chicken (16.9 ± 0.2 nL/min/mg protein) followed by geese (5.0 ± 0.6 nL/min/mg protein). Meanwhile, similar clearance rates of clothianidin metabolite were found in ducks (1.6 ± 0.1 nL/min/mg protein), quails (2.4 ± 0.5 nL/min/mg protein), and rats (1.6 ± 0.2 nL/min/mg protein). Human CYP2B6, CYP2C19, and CYP3A4 are known to be involved in the conversion of thiamethoxam to clothianidin [16]. Pan et al. [19] reported that the metabolism of thiamethoxam in quails is predominantly mediated by CYP and glutathione metabolism pathways. In addition, molecular dynamic simulation showed the strongest binding interaction between quail CYP2H1 and thiamethoxam [19]. CYP2H genes were assumed to be avian-specific and corresponded to CYP2C genes in mammals [20]. Thus, our study revealed that chicken CYP3A and CYP2H may have a higher potential to metabolize thiamethoxam substrate to clothianidin compared to rats.

### 3.2. Higher Capacity for CYP-Dependent Metabolism of Neonicotinoids in Chickens

The interspecies differences in metabolite formations and CYP-mediated metabolism of neonicotinoids were observed in domestic poultry. Katagi and Fujisawa [18] reviewed that the total amount and activity of avian CYPs depend on species; they may also vary by sex, age, and strain. Among the four target poultry species, chickens showed a specific metabolism of acetamiprid and thiamethoxam by microsomes containing CYPs. From the LC/Q-TOF analysis, IM-1-5 (acetamiprid metabolite) was detected in microsomes of all target species of this study except in that of chickens; in sharp contrast, however, the dm-clothianidin metabolite of clothianidin was found only in the microsome reactions of chicken. In addition, kinetic parameters of neonicotinoid metabolism indicated that the maximum rates of oxidations of acetamiprid to dm-acetamiprid; imidacloprid to 4OH-imidacloprid, 5OH-imidacloprid, and olefin; clothianidin to dm-clothianidin, and thiamethoxam to clothianidin were found in chickens. Our previous finding also suggested that the intrinsic hepatic clearance of fipronil and the capacity of CYPs to metabolize fipronil to sulfone was most efficient in chickens compared to other birds [27]. These findings indicated that microsomes of chickens may have greater capacity for CYP-dependent metabolism of neonicotinoids compared to ducks, geese, and quails. However, future studies are required to elucidate the specific CYP isoforms of neonicotinoid metabolism in poultry species using CYP recombinant enzymes. In addition to CYPs, further investigations on other phase I and II metabolism enzymes related to neonicotinoid metabolism such as aldehyde oxidase and glutathione S-transferase are required.

## 4. Conclusions

In conclusion, this study was the first elucidation on the specific compositions of metabolites and the interspecies differences in CYP-mediated metabolism of several neonicotinoids in poultry using liver microsomes. Microsomes of chicken may contain higher potential CYPs to metabolize neonicotinoid substrates to various major metabolites, causing the specific formations of metabolites found in chicken compared to ducks, geese, quails, and rats. CYPs in ducks may effectively associate with the nitro-reduction of imidacloprid and clothianidin by showing the highest V_max_/K_m_ values of dn-imidacloprid and dn-clothianidin in this study. These findings supported the hypothesis that species and individual substrates could influence the variance of CYP-dependent neonicotinoid metabolism using in vitro microsomal assays.

## Figures and Tables

**Table 1:** Neonicotinoid metabolites identified by LC/Q-TOF in reaction mixtures using liver microsomes from various species.

Substrate	Metabolite	Formula	RT (min)	Mass	Mass (Target)	Mass Difference (ppm)	Score	Species
Acetamiprid	*N*-desmethyl-acetamiprid	C_9_H_9_ClN_4_	12.9	208.0514–208.0517	208.0516	−0.81–0.55	98.4–99.7	Chicken, Duck, Goose, Quail, Rat
	*N*-methyl(6-chloro-3-pyridyl) methylamine	C_7_H_9_ClN_2_	5.5	156.0446–156.0452	156.0454	−5.16–(−1.14)	77.2–99.5	Chicken, Duck, Goose, Quail, Rat
	*N*-[(6-chloro-3-pyridyl) methyl]-*N*-methyl acetamidine	C_9_H_12_ClN_3_	4.5	197.0709–197.0738	197.0720	−5.32–9.28	51.1–66.8	Duck, Goose, Quail, Rat
Imidacloprid *	4OH- and/or 5OH-imidacloprid	C_9_H_10_ClN_5_O_3_	10.7	271.0468–271.0471	271.0472	−1.59–(−0.44)	92.9–96.5	Chicken, Duck, Rat
	dn-imidacloprid	C_9_H_11_ClN_4_	7.8	210.067–210.0671	210.0672	−1.32–(−0.45)	59.1–97.4	Chicken, Duck, Rat
	imidacloprid-olefin	C_9_H_8_ClN_5_O_2_	10.5	253.0363–253.0364	253.0367	−1.02–(−0.67)	88.1–96.3	Chicken, Duck, Rat
Clothianidin *	dm-clothianidin	C_5_H_6_ClN_5_O_2_S	10.6	234.9928–234.9929	234.9931	−1.25–(−0.57)	95.3–97.5	Chicken, Duck, Rat
	clothianidin-urea	C_6_H_8_ClN_3_OS	9.9	205.0073–205.0074	205.0077	−1.88–(−1.05)	94.9–99.5	Chicken, Duck, Rat
Thiamethoxam	clothianidin	C_6_H_8_ClN_5_O_2_S	11.8	249.0084–249.0087	249.0087	−1.15–0.02	98.9–99.5	Chicken, Duck, Goose, Quail, Rat
	clothianidin-urea	C_6_H_8_ClN_3_OS	9.9	205.0065–205.0084	205.0077	−5.49–3.8	67.5–90.8	Chicken, Duck, Goose, Quail, Rat
	dm-clothianidin	C_5_H_6_ClN_5_O_2_S	10.6	234.9924–234.9928	234.9931	−2.96–(−1.03)	59.1–73.5	Chicken
	*N*-desmethyl-thiamethoxam	C_7_H_8_ClN_5_O_3_S	12.3	277.0029–277.0036	277.0036	−3.07–(−0.49)	85.5–99.1	Chicken, Duck, Goose, Quail, Rat
	thiamethoxam-urea	C_8_H_10_ClN_3_O_2_S	13.1	247.0177–247.018	247.0182	−2.27–(−0.73)	93.8–98.6	Chicken, Duck, Goose, Quail, Rat

* The substrate was not analyzed using microsomes of goose and quail.

**Table 2:** Comparison of Michaelis–Menten kinetic parameters (V_max_ (pmol/min/mg protein), K_m_ (μM), V_max_/K_m_, (nL/min/mg protein), mean ± SD) for CYP metabolism of neonicotinoids among various species.

Substrate	Metabolite	Parameter	Species				
			Chicken	Duck	Goose	Quail	Rat
Acetamiprid	dm-acetamiprid	V_max_	1.2 ± 0.0	0.4 ± 0.0	1.3 ± 0.2	0.3 ± 0.1	0.1 ± 0.0
		K_m_	25.6 ± 1.4	67.5 ± 9.9	48.2 ± 8.0	35.3 ± 10.3	55.8 ± 8.7
		V_max_/K_m_	46.5 ± 3.5 ^a^	5.7 ± 0.4 ^c^	27.7 ± 0.5 ^b^	7.2 ± 0.2 ^c^	1.5 ± 0.1 ^c^
Imidacloprid	4OH-imidacloprid	V_max_	0.5 ± 0.0	0.1 ± 0.0	-	-	0.1 ± 0.0
		K_m_	31.2 ± 3.0	77.9 ± 19.1			138.5 ± 120.8
		V_max_/K_m_	17.1 ± 0.6 ^a^	1.6 ± 0.1 ^b^			1.1 ± 0.3 ^b^
	5OH-imidacloprid	V_max_	0.5 ± 0.0	0.1 ± 0.0	-	-	0.1 ± 0.0
		K_m_	34.1 ± 0.6	87.4 ± 27.7			112.0 ± 56.1
		V_max_/K_m_	15.3 ± 0.0 ^a^	0.8 ± 0.1 ^b^			0.8 ± 0.1 ^b^
	dn-imidacloprid	V_max_	1.0 ± 0.7	0.2 ± 0.0	-	-	0.1 ± 0.0
		K_m_	699.5 ± 500.0	37.6 ± 8.2			26.2 ± 0.7
		V_max_/K_m_	1.6 ± 0.2 ^b^	5.5 ± 0.6 ^a^			2.8 ± 0.1 ^b^
	imidacloprid-olefin	V_max_	0.2 ± 0.0	0.03 ± 0.0	-	-	NF
		K_m_	29.4 ± 7.9	42.2 ± 0.0			
		V_max_/K_m_	7.5 ± 0.9 ^a^	0.8 ± 0.1 ^b^			
Clothianidin	dm-clothianidin	V_max_	4.4 ± 1.2	2.9 ± 1.3	-	-	NF
		K_m_	52.0 ± 21.8	358.8 ± 182.2			
		V_max_/K_m_	86.8 ± 12.6 ^a^	8.1 ± 0.4 ^b^			
	dn-clothianidin	V_max_	0.1 ± 0.0	0.3 ± 0.1	-	-	0.2 ± 0.0
		K_m_	27.5 ± 18.4	29.1 ± 8.2			39.9 ± 9.7
		V_max_/K_m_	3.0 ± 1.3 ^b^	10.9 ± 0.2 ^a^			5.5 ± 1.0 ^b^
	clothianidin-urea	V_max_	0.008 ± 0.001	0.005 ± 0.001	-	-	0.012 ± 0.002
		K_m_	119.9 ± 24.1	75.2 ± 26.6			209.6 ± 56.6
		V_max_/K_m_	0.07 ± 0.01	0.07 ± 0.02			0.06 ± 0.00
Thiamethoxam	clothianidin	V_max_	1.2 ± 0.0	0.1 ± 0.0	0.2 ± 0.1	0.4 ± 0.1	0.1 ± 0.0
		K_m_	73.4 ± 0.1	92.7 ± 20.9	48.3 ± 19.6	181.5 ± 82.7	63.0 ± 20.0
		V_max_/K_m_	16.9 ± 0.2 ^a^	1.6 ± 0.1 ^c^	5.0 ± 0.6 ^b^	2.4 ± 0.5 ^c^	1.6 ± 0.2 ^c^
	dm-clothianidin	V_max_	0.01 ± 0.00	ND	ND	ND	ND
		K_m_	13.0 ± 0.8				
		V_max_/K_m_	0.4 ± 0.0				

Different characters (^a^, ^b^, and ^c^) indicate statistically significant differences of V_max_/K_m_ (Tukey–Kramer test, *p* < 0.05); NF, not fit to Michaelis–Menten plot; ND, not detected; -, The substrate was not analyzed using microsomes of goose and quail.

## Data Availability

The original data presented in this study are included in the article. Further inquiries can be directed to the corresponding author.

## Supplementary Materials

The following supporting information can be downloaded at: https://www.mdpi.com/article/10.3390/toxics12080618/s1, Table S1: Neonicotinoids and target metabolites for LC/MS/MS analysis; Table S2: Details of studied animals to extract liver microsomes.; Table S3: Screening neonicotinoids and target metabolites using LC/Q-TOF.
