# Supplementary material for: Characterization of Neonicotinoid Metabolites by Cytochrome P450-Mediated Metabolism in Poultry
Source: Toxics. 2024 Aug 21;12(8):618. doi: 10.3390/toxics12080618 (PMC11359332; doi:10.3390/toxics12080618)
Supplement: Supplementary file 1 [file toxics-12-00618-s001.zip › toxics-3115955-supplementary.pdf]

Table S1. Neonicotinoids and target metabolites for LC/MS/MS analysis.

Target Compound	Retention Time (min)	MRM (*m*/*z*)	CE (V)	Polarity	Source of Standard Solution
Acetamiprid	11.64	223.1 > 126.00	21	+	Wako Pure Chemical
6-CNA	5.78	158.00 > 121.90	17	+	Sigma-Aldrich
dm-acetamiprid	9.23	209.1 > 126.00	16	+	Sigma-Aldrich
Imidacloprid	10.46	256.1 > 175.00	18	+	Wako Pure Chemical
4OH-imidacloprid	8.9	272.00 > 225.00	16	+	Toho University
5OH-imidacloprid	8.4	272.00 > 228.00	8	+	Toho University
6-CNA	6.56	158.00 > 122.00	19	+	Sigma-Aldrich
dn-imidacloprid	6.62	211.1 > 126.00	26	+	Sigma-Aldrich
Imidacloprid-olefin	8.87	254.00 > 236.00	4	+	Sigma-Aldrich
Clothianidin	8.5	250.00 > 169.1	7	+	Wako Pure Chemical
Clothianidin-urea	5.4	192.00 > 175.2	16	+	Toho University
dm-clothianidin	7.4	236.00 > 131.9	7	+	Toho University
dm-clothianidin-urea	6.8	206.00 > 131.9	29	+	Toho University
dm-dn-clothianidin	3.4	191.00 > 132.1	18	+	Toho University
dn-clothianidin	4.2	205.00 > 132.1	22	+	Toho University
Thiamethoxam	8.91	292.00 > 211.00	6	+	Wako Pure Chemical
6-CNA	6.56	158.00 > 122.00	19	+	Sigma-Aldrich
Clothianidin	8.23	250.00 > 169.1	9	+	Wako Pure Chemical
dm-clothianidin	7.18	236.00 > 131.9	13	+	Toho University

Table S2. Details of studied animals to extract liver microsomes.

Species	Chicken	Duck	Goose	Quail	Rat
**Scientific name**	*Gallus domesticus*	*Anas platyrhynchos*	*Anser domesticus*	*Coturnix coturnix*	*Rattus norvegicus*
**Age**	1 year	1.5 years	1.5 years	8 months	8 weeks
**Sex**	Male	Female	Male	Male	Male
**Number of samples**	5	5	3	5	3

Table S3. Screening neonicotinoids and target metabolites using LC/Q-TOF

Parent compound	Target metabolite	Formula	CAS	Source
Imidacloprid	Imidacloprid	C9H10ClN5O2	138261-41-3	https://efsa.onlinelibrary.wiley.com/doi/pdf/10.2903/j.efsa.2019.5570
	Imidacloprid-5-hydroxy	C9H10ClN5O3	1115248-02-6	https://efsa.onlinelibrary.wiley.com/doi/pdf/10.2903/j.efsa.2019.5570
	Imidacloprid olefin	C9H8ClN5O2	115086-54-9	https://efsa.onlinelibrary.wiley.com/doi/pdf/10.2903/j.efsa.2019.5570
	Imidacloprid desnitro	C9H11ClN4	115970-17-7	https://efsa.onlinelibrary.wiley.com/doi/pdf/10.2903/j.efsa.2019.5570
	Imidaloprid-6-CNA	C6H4ClNO2	5326-23-8	https://efsa.onlinelibrary.wiley.com/doi/pdf/10.2903/j.efsa.2019.5570
	Imidacloprid-CHMP	C6H6ClNO	21543-49-7	https://efsa.onlinelibrary.wiley.com/doi/pdf/10.2903/j.efsa.2019.5570
	4,5-Dihydroxy imidacloprid	C9H10ClN5O4	155802-65-6	https://link.springer.com/article/10.1007/s11356-014-3470-y
Acetamiprid	Acetamiprid	C10H11ClN4	135410-20-7	https://efsa.onlinelibrary.wiley.com/doi/epdf/10.2903/j.efsa.2022.7535
	N-desmethyl-acetamiprid	C9H9ClN4	190604-92-3	https://efsa.onlinelibrary.wiley.com/doi/epdf/10.2903/j.efsa.2022.7535
	N-methyl(6-chloro-3-pyridyl) methylamine	C7H9ClN2	120739-62-0	https://efsa.onlinelibrary.wiley.com/doi/epdf/10.2903/j.efsa.2022.7535
	N-[(6-chloro-3-pyridyl)methyl]-*N*-methyl acetamidine	C9H12ClN3	365441-66-3	https://efsa.onlinelibrary.wiley.com/doi/epdf/10.2903/j.efsa.2022.7535
	6-chloronicotinic acid	C6H4ClNO2	5326-23-8	https://efsa.onlinelibrary.wiley.com/doi/epdf/10.2903/j.efsa.2022.7535
	6-chloropicolyl alcohol	C8H7ClO3	2591-25-5	https://link.springer.com/article/10.1007/s11356-014-3470-y
Clothianidin	Clothianidin	C6H8ClN5O2S	210880-92-5	https://pubs.acs.org/doi/10.1021/tx0601859
	Desmethyl-clothianidin	C5H6ClN5O2S	135018-15-4	https://pubs.acs.org/doi/10.1021/tx0601859

	Nitroguanidine	CH4N4O2	556-88-7	https://pubs.acs.org/doi/10.1021/tx0601859
	Clothianidin-urea	C6H8ClN3OS	634192-72-6	https://pubs.acs.org/doi/10.1021/tx0601859
	1-Methyl-3-nitroguanidine	C2H6N4O2	4245-76-5	https://pubs.acs.org/doi/10.1021/tx0601859
Thiamethoxam	Thiamethoxam	C8H10ClN5O3S	153719-23-4	https://pubs.acs.org/doi/10.1021/tx0601859
	N-desmethyl-thiamethoxam	C7H8ClN5O3S	171103-04-1	https://pubs.acs.org/doi/10.1021/tx0601859
	Thiamethoxam-urea	C8H10ClN3O2S	902493-06-5	https://pubs.acs.org/doi/10.1021/tx0601859
	Clothianidin	C6H8ClN5O2S	210880-92-5	https://pubs.acs.org/doi/10.1021/tx0601859
	Desmethyl-clothianidin	C5H6ClN5O2S	135018-15-4	https://pubs.acs.org/doi/10.1021/tx0601859
	Nitroguanidine	CH4N4O2	556-88-7	https://pubs.acs.org/doi/10.1021/tx0601859
	Clothianidin-urea	C6H8ClN3OS	634192-72-6	https://pubs.acs.org/doi/10.1021/tx0601859
